# Gene editing using CRISPR-Cas9 for the treatment of lung cancer

**Published:** 2016-12-30

**Authors:** Andres Castillo

**Affiliations:** 1 Profesor Asistente, Departamento de Biologia, Facultad de Ciencias Naturales y Exactas. Universidad Del Valle. Cali, Colombia; 2 Editor Asociado, Revista Colombia Médica, Universida del Valle,Cali, Colombia

In 2015, the journal Science chose CRISPR-Cas9 technology as the most important technological advance of science in the last years ^1^. This magazine announced the beginning of a new era of biotechnology in which it would be possible to edit, correct and modify the genetic information of any cell in a feasible, fast and cheap way; and most importantly, with high precision. Its implementation in research laboratories in basic and applied sciences could help to develop therapeutic strategies for the health area with the main objective of healing diseases with a known genetic origin, and that until now have been impossible to cure.

**Figure 1:**
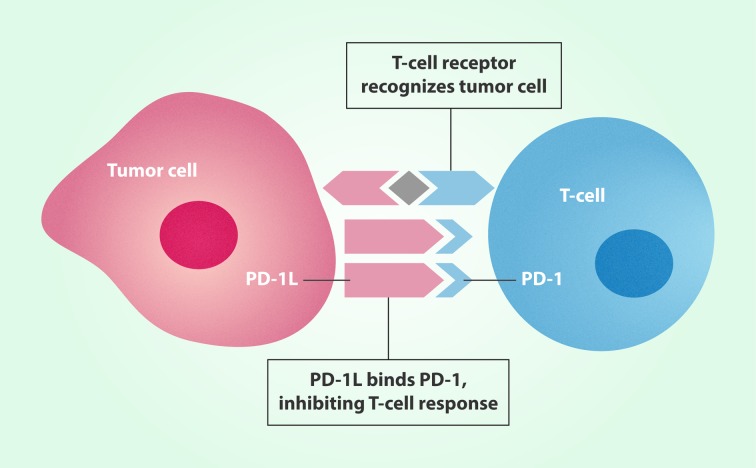
Tumor cells can inhibit body´s immune response by binding to proteins, such as PD-1, on the surface of T cells. CRISPR-Cas9 technology that turn off PD-1 gene reactivate the immune response.

It was not long since this announcement when Nature magazine surprised the scientific community by publishing, on November 15, 2016, that researchers from the University of Sichuan, China, had been able to inject genetically modified lymphocytes for the first time to a patient with lung cancer as a therapeutic approach to promote the immune system's response for eliminating malignant tumor cells ^2^. To achieve this goal, the gene coding for the PD-1 protein (for "Programmed Death-1") was switched off using CRISPR-Cas9 technology, so it is expected that the action of the immune system mediated by lymphocytes against cancer be more effective (Fig. 1). Even though there are already approved treatments for lung cancer where PD-1 protein is blocked by immunotherapy, it is expected that PD-1 gene inactivation be a therapeutic strategy with greater efficiency and stability.

But what is this new technological breakthrough called CRISPR-Cas9, and how does it inactivate cancerous mutations? The history of CRISPR-Cas9 began in 1987, when a group of Japanese scientists from the University of Osaka, Japan, studying the genetic information present in bacteria, reported the finding of repeated DNA sequences, apparently without any function ^3^. Years later, in 1993, the team of researcher Juan Francisco Martínez Mojica of the University of Alicante, Spain, independently reported the same finding but in archaeal genomes, and described the sequences as repetitive and palindromic, which were separated from each other by means of spacer sequences, and which had a leader sequence at their start ^4^. Martinez *et al.* called these sequences CRISPRs, acronym for "Clustered Regularly Interspaced Short Palindromic Repeats" ^5^.

When analyzing the CRISPR sequences, the researchers realized their similarity to sequences of bacteriophages and plasmids; besides the fact that they were located near *cas* genes, which coded for a type of nucleases capable of cutting and degrading exogenous DNA in specific sequence sites. The above led to conclude that CRISPR sequences could be part of a novel prokaryotic defense system against the invasion of viral agents and plasmids. This system is composed by a Cas protein linked to an RNA coming from the CRISPR sequences, which was called the CRISPR-Cas complex; this complex is activated by the presence of foreign DNA from invasive bacteriophages or plasmids, and it is able to recognize and degrade that foreign DNA ^6^. 

These observations were later verified with the finding that the Cas proteins that made up the complex were not only capable of cutting foreign DNA but could also integrate a small fragment of the foreign DNA digested within the CRISPR sequences, and this way acquire an immune memory for future attacks by the same type of virus. Thus, the CRISPR-Cas complex is actually an adaptive immune defense system of the prokaryotes, which they can transmit to their offspring ^7^ ^,^ ^8^.

**Figure 2:**
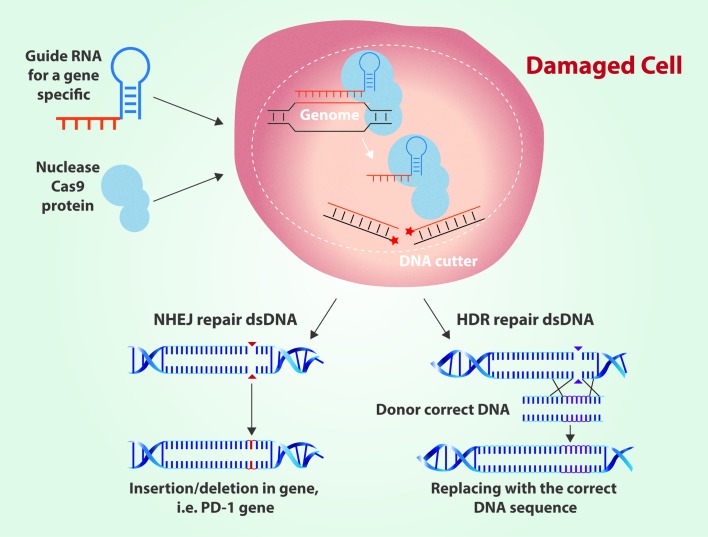
Gene editing by CRISPR-Cas9 using Non-homologous end joining repair (NHEJ) and Homology directed repair (HDR).

Years later, in 2012, a team of researchers led by Jennifer Doudna, of the University of California, at Berkeley, and Emmanuelle Charpentier, of the University of Umea, proposed the possibility of modifying and implementing the CRISPR-Cas complex to apply it as a biotechnological tool for editing "programmable" genomes; that a DNA strand could be specifically cut *in vitro* for therapeutic purposes ^9^. To be able to carry out this idea, the researchers generated a protocol that consisted of three basic steps. The first one consists of the design and synthesis of a single-stranded RNA molecule, called guiding RNA, which can bind to the Cas9 enzyme, and which complies with the same molecular characteristics of the CRISPR sequences, *i.e.*, it is capable of recognizing and joining to a specific sequence of DNA or gene, which it is wanted to be edited or corrected. Once obtained the guide RNA bound to Cas9, the second step is to introduce *in vitro* the CRISPR-Cas9 complex into the cell to be treated, and once inside, the complex will recognize the exact site of the genome where the Cas9 enzyme must cut (Fig. 2). 

The third step begins with the activation of the cell repair mechanisms in response to the cut that was performed by the Cas9 endonuclease, which may cause the loss or addition of genetic information in the region of the genome to be edited. This can lead to the loss of the original function of the gene, resulting in an inactivation or malfunction of the protein it encodes. If the goal is to replace the cancerous mutations, to this last step can be incorporated some changes for correct genetic information. This procedure consists in adding a homologous template DNA molecule of the region to be edited and that does not contain the carcinogenic mutations. Activating a repair system by homologous recombination, it can be replaced the DNA fragment previously recognized and treated by the complex CRIPR-Cas9 ^10^. 

One the objectives of the study published by Nature, where it will be evaluated whether the treatment with CRISPR-Cas9 technology is effective against lung cancer, is to test the safety of the assay for patients ^11^. In the clinical trial, they intend to treat only 10 patients with 3 different dose regimens, which will be followed in order to monitor some type of adverse effect in the modified lymphocytic cells. In the phase I of the clinical trial being developed by the scientific team of Dr. Lu You, an oncologist at Sichuan University's West China Hospital, there will participate patients with non small cell lung cancer with metastases, which had a recurrence of cancer after initial therapy or were refractory to chemotherapy after treatment. In the procedure, lymphocytes were extracted from the patients' blood to be treated with the CRISPR-Cas9 genomic editing system, in order to disable the gene encoding the PD-1 protein. Lymphocytic cells, inhibited for the expression of the PD-1 gene, were tested for their viability and lymphoproliferation to rule out new mutations during treatment. Next, the lymphocytes were transfused to a single patient, whom is being monitored while waiting for the modified cells to reach the tumor tissue and activate an immune response against the cancer cells. Among the risks that exist for therapeutic treatment with CRIPR-Cas9 technology is that an excessive autoimmune response may occur. So far, the available information on the progress of the project is positive, as the patient has not presented adverse effects; besides, it is planned to inject a second dose of modified lymphocytes, so we must still wait for the final results of the pioneering study on CRISPR- Cas9 for the inhibition of genes in cancer patients. Likewise, additional studies should be carried out to evaluate the safety of the technology and the possible occurrence of adverse effects.

By now, CRISPR-Cas9 gene-editing technology is the best bet to achieve an effective therapeutic cure for diseases of genetic origin, such as some cancers; but in the future, the development of these tools will increase its potential uses and the population that it can benefit. For this reason, the development of ethical guidelines for the investigation and clinical application of human gene editing is a priority, in order to prevent ethical risks with the use of these tools. In particular, further study is needed on the risks of gene editing for eugenic purposes, or to generate inequity by improving physical, intellectual or cosmetic characteristics and the transmission to the offspring of the edited genes. Ethics need to be discussed by distinguishing between gene-editing research in somatic cells, germ cells, or human embryos.

In this sense, the scientific community held in December 2015 an international meeting called "International Summit on Human Gene Editing" ^12^, in which had participation the academies of science, engineering and medicine of the United States, the United Kingdom, and China. In that meeting, there were discussed, the precautions that must be taken for the implementation of CRISPR-Cas9 technique in human research. Attending the meeting were the proponents of the technique and scientists who had applied the technique to human embryos; besides, there were scientists and philosophers with bioethical training, and lawyers who were knowledgeable about patent laws. The most important conclusions of the event were the need to establish legal, ethical and follow-up standards for basic and preclinical research on human gene expression, and the use of the technique in somatic cells for clinical applications; and with some restrictions, in germ-line cells or embryos, at least until sufficient information is obtained on the safety and efficacy of the same. In this last point, it was recommended to continue the discussion and standardization of the norms referring to an acceptable use of the techniques of genes editing in the human germinal line for therapeutic purposes worldwide. Recently, however, the ethics committee of the Biomedical Research Agency of France (INSERM) set out its position about it and recommended the prohibition of all genetic modifications of the germline ^13^. It also proposed the creation of a European committee of experts from different disciplines to evaluate the scope, effectiveness and safety of CRISPR-Cas9, and a follow-up group of the involved parts in order to promote an open debate on the social aspects of these technologies. 
